# 3-Nitrooxypropanol (Feed Additives)

**DOI:** 10.14252/foodsafetyfscj.D-24-00008

**Published:** 2024-06-28

**Authors:** Food Safety Commission of Japan

## Abstract

Food Safety Commission of Japan (FSCJ) conducted a risk assessment of 3-nitrooxypropanol (3-NOP) (CAS No. 100502-66-7), using the evaluation documents for feed additive designation. This feed additive is a nitrate ester of 1,3-propanediol developed to reduce methane generated in the first stomach of cattle (rumen). The data used in the assessment include the fate in animals (mice, rats, cattle), residues (cattle), genotoxicity, acute toxicity (rats), subacute toxicity (mice, rats and dogs), chronic toxicity/carcinogenicity (rats), reproductive/developmental toxicity (rats and rabbits), and others. The lowest no-observed-adverse-effect level (NOAEL) for possible adverse effects of 3-NOP was 100 mg/kg bw per day in 52-chronic toxicity, 104-week chronic toxicity/carcinogenicity, and two-generation reproductive toxicity studies in rats. Given this, FSCJ specified an acceptable daily intake (ADI) of 1 mg/kg bw per day by applying a safety factor of 100 to the NOAEL.

## Conclusion in Brief

Food Safety Commission of Japan (FSCJ) conducted a risk assessment of 3-nitrooxypropanol (3-NOP) (CAS No. 100502-66-7), using the evaluation documents for feed additive designation. This feed additive is a nitrate ester of 1,3-propanediol developed to reduce methane generated in the first stomach of cattle (rumen).

The data used in the assessment include the fate in animals (mice, rats, cattle), residues (cattle), genotoxicity, acute toxicity (rats), subacute toxicity (mice, rats and dogs), chronic toxicity/carcinogenicity (rats), reproductive/developmental toxicity (rats and rabbits), and others.

In studies of residues and fate in animals, 3-NOP was metabolized rapidly to NOPA (M2)*^1^, HPA (M7)*^2^ in the ruminants, and the parent substance was not detected in the plasma about 1-2 hours after the ingestion. These metabolites were also identified in rats. M2 as well as 3-NOP were recognized as the relevant substances in human consuming livestock, due to the exceeding amounts of M2 over residual 3-NOP. Further, metabolites such as M7 were detected normally in living organism and thus were judged not to have safety concern.

An *in vitro* micronucleus test for 3-NOP indicated the positive results in a genotoxicity study. All other test results including an *in vivo* micronucleus test showed negative. An *in vitro* reverse mutation test showed positive results, while all the other tests including genetic gene mutation test with transgenic rats were negative for a metabolite NOPA. Thereby FSCJ judged that 3-NOP and NOPA would not cause genotoxicity for living organism.

The following results were observed in the subacute toxicity study, 1) Decrease in the absolute and relative weights of male testis and epididymis, 2) Decrease in sperm counts, 3) Decrease in the motor activity of the sperm.

No genotoxic potential was suggested on 3-NOP genotoxicity study as indicated above, though benign mesenchymal tumor was observed in the duodenum and the jejunum in the carcinogenicity study. FSCJ thought that threshold setting would be possible for the assessment.

In the two-generation reproductive toxicity study in rats, the toxicity effects were not observed for the group of dams and offspring at doses up to 100 mg/kg bw per day on the general conditions, various clinical findings, and also effects on fertility. No teratogenicity was observed in the developmental toxicity study.

The testicular toxicity of 3-NOP to rats was reasonably expected due to the metabolite NOPA but not to HPA. The study on fate of 3-NOP suggested the ultimate toxicity of NOPA, since 3-NOP was rapidly metabolized in the body.

The lowest no-observed-adverse-effect level (NOAEL) for possible adverse effects of 3-NOP was 100 mg/kg bw per day in 52-chronic toxicity, 104-week chronic toxicity/carcinogenicity, and two-generation reproductive toxicity studies in rats. Given this, FSCJ specified an acceptable daily intake (ADI) of 1 mg/kg bw per day by applying a safety factor of 100 to the NOAEL.

## References

[1] Food Safety Commission of Japan. Risk Assessment Report. 3-Nitrooxypropanol (Feed Additives) [in Japanese]. https://www.fsc.go.jp/fsciis/attachedFile/download?retrievalId=kya20230309035&fileId=201. 10.14252/foodsafetyfscj.D-24-00008 PMC11257681 39036746

